# Loss of Environmental Enrichment Elicits Behavioral and Physiological Dysregulation in Female Rats

**DOI:** 10.3389/fnbeh.2018.00287

**Published:** 2019-01-21

**Authors:** Rachel Morano, Olivia Hoskins, Brittany L. Smith, James P. Herman

**Affiliations:** Department of Pharmacology and Systems Physiology, University of Cincinnati, Cincinnati, OH, United States

**Keywords:** anhedonia, coping behavior, corticosterone, estrus cycle, sex differences, stress

## Abstract

Chronic stress drives behavioral and physiological changes associated with numerous psychiatric disease states. In rodents, the vast majority of chronic stress models involve imposition of external stressors, whereas in humans stress is often driven by internal cues, commonly associated with a sense of loss. We previously exposed groups of rats to environmental enrichment (EE) for a protracted period (1 month), followed by removal of enrichment (ER), to induce an experience of loss in male rats. ER enhanced immobility in the forced swim test (FST), led to hypothalamic pituitary adrenal (HPA) axis hypoactivity, and caused hyperphagia relative to continuously enriched (EE), single-housed (Scon) and pair-housed (Pcon) groups, most of which were reversible by antidepressant treatment (Smith et al., 2017). Here, we have applied the same approach to study enrichment loss in female rats. Similar to the males, enrichment removal in females led to an increase in the time spent immobile in the FST and increased daytime food intake compared to the single and pair-housed controls. Unlike males, ER females showed decreased sucrose preference, and showed estrus cycle-dependent HPA axis hyperactivity to an acute restraint stress. The increase in passive coping (immobility), anhedonia-like behavior in the sucrose preference test and HPA axis dysregulation suggest that enrichment removal produces a loss phenotype in females that differs from that seen in males, which may be more pronounced in nature.

## Introduction

The experience of loss represents a significant risk factor for affective distress and disease. Life events connected with loss of financial resources, loved ones, relationships or liberties are among the most often reported experiences linked to negative mental health outcomes, prominently including depression and anxiety disorders (Rahe, 1968; Ganzini et al., 1990; Sikorski et al., 2014; Wang et al., 2015; Wiseman et al., 2015). However, there have been relatively few studies focusing on neurobiological mechanisms underlying the loss of positively rewarding life experiences.

Our lab has recently developed an enrichment removal model for induction of lasting behavioral and physiological responses to loss. In males, prolonged exposure to enrichment followed by removal to single housing results in reproducible increases in passive coping behavior, weight gain, and hypothalamic-pituitary-adrenocortical (HPA) axis hypoactivity that is not emulated by single housing alone, chronic restraint exposure or chronic variable stress, indicating that the behavioral and somatic phenotype is unique to experiencing cessation of enrichment. Moreover, antidepressant treatment prevented the symptoms of enrichment removal, suggesting a linkage to biological/emotional processes regulated by this class of drug (Smith et al., 2017).

Work on the fundamental biology of stress indicates massive sex differences in behavioral and physiological endpoints. While the field is not in universal agreement on the nature and extent of sex differences, in general work suggests that chronic imposed stress (e.g., chronic mild stress, chronic variable stress, chronic social defeat) reliably affects coping behavior [e.g., increased immobility in the forced swim test (FST)], anhedonia and physiology (e.g., increased HPA axis reactivity) (Willner et al., 1987; Herman et al., 1995; Rygula et al., 2005, 2008; Kompagne et al., 2008). There is some evidence to suggest that females are more vulnerable to chronic mild stress, as demonstrated by decreased sucrose preference, increased immobility in the FST, decreased open field activity and more pronounced HPA axis responses to stress (Dalla et al., 2005; Xing et al., 2013; Lu et al., 2015; Rincon-Cortes and Grace, 2017). The enhanced stress phenotype seen in rats is in keeping with human reports indicating a nearly 2:1 incidence of stress-related diseases (depression, PTSD) in females relative to males (Kessler et al., 1993; Olff et al., 2007), suggesting that being female is a significant risk factor. Consequently, it is important to understand the impact of experience of loss on behavioral and physiological reactivity in the female. Therefore, the current study explored the impact of ER in females in light of what we know from males, focusing on behavioral endpoints showing the most consistent and pronounced male phenotypes. Our data suggest that while males and females share core features of enrichment removal, others (e.g., HPA axis reactivity and sucrose preference) differ substantially.

## Materials and Methods

### Animals

Eighty female Sprague-Dawley rats, weighing between 180–220 g, were obtained from Harlan (Indianapolis, IN, United States) at approximately 9 weeks of age. Upon arrival, rats were randomly assigned to a housing manipulation and cohort. From the eighty rats, we created two cohorts, with each cohort containing forty rats. All rats were housed with corncob bedding and *ad libitum* water and chow (3.41 kcal/g, 0.51 kcal/g from fat; Harlan Teklad, Madison, WI, United States). The vivarium had a 12 h light:dark cycle (9am lights on, 9pm lights off) and was temperature (23°C) and humidity (50%) controlled. All procedures were conducted in compliance with the National Institutes of Health guidelines for the Care and Use of Animals, and were approved by the University of Cincinnati Institutional Animal Care and Use Committee.

### Experimental Groups

**FIGURE 1:**
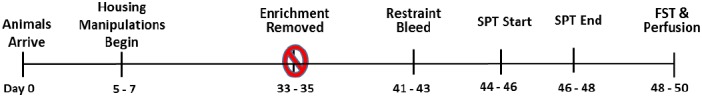
Experimental time-line.

Animals were divided into two cohorts to optimize behavioral testing (see Supplementary Table S1). Decreasing the number of animals tested per day allowed for the testing time to be done closer to the circadian corticosterone trough and thus minimizing the fluctuations in diurnal hormone levels at the time of testing (Atkinson and Waddell, 1997) Cohort 1 experienced 5 days of acclimation to the vivarium, and cohort 2 experienced 7 days. Each cohort underwent the same experimental timeline and protocols 2 days apart (Figure 1). Animals received housing manipulations during the 12 h dark (active) cycle only (Smith et al., 2017). Housing manipulations began for cohort 1 at lights out of day 5 and began 2 days later for cohort 2 at lights out of day 7. Each cohort consisted of four treatment groups, with treatment (housing manipulation) occurring during the dark (active) cycle only. Groups included single-housed animals (Scon *n* = 10 per cohort, *n* = 20 total); pair-housed control animals (Pcon *n* = 10 per cohort, *n* = 20 total); continuously enriched animals (EE *n* = 10 per cohort, *n* = 20 total); and enrichment-removed animals (ER *n* = 10 per cohort, *n* = 20 total). With the exception of the enrichment-removed group, all active cycle housing manipulations continued until the end of the experiment (Figure 1). Animals removed from enrichment were removed after 4 weeks. ER animals were given a week of single-housing during active cycle before behavioral testing began, to ensure a phenotype had time to develop (Smith et al., 2017).

For all experiments, the Pcon group serves as the control group for comparison of ER, EE and Scon manipulations. The Scon group evaluates the impact of isolation alone on the noted experimental endpoints.

### Housing Manipulations

Standard housing for all rats during the inactive cycle was single-housing (home cage), which consisted of one rat per polycarbonate shoebox cage (20 cm H × 22 cm W × 43 cm L). Use of a home cage during the inactive cycle allowed for data collection from individuals. Animals that were single-housed during the active cycle remained in home cages but were handled briefly twice daily at the time when other animals were moving between housing environments to account for the stress of cage disruption (Sharp et al., 2003) and interactions with the researcher. Animals in the pair housed group were only pair housed during the active period (lights off), and the pair housing was done in a cage that was not the home cage of either animal in the pair. Cages used for pair housing were identical to the home cage. Cage mates remained constant throughout the experiment. Animals experiencing enrichment were moved to enrichment cages within a separate room during the dark cycle and received crinkle paper in the home cage when single-housed during the light cycle. 1 h before lights out and within 1 h of lights being on, all animals were handled in order to both transfer cages and to habituate the animals to interactions with the researcher.

Four enrichment cages (1 m H × 1 m W × 1 m L) were adorned with a large metal feeding bowl, three standard water bottles, and corncob bedding. One group (*n* = 10) was housed per enrichment cage. Each cage received 3–5 novel objects in addition to crinkle paper, metal ladders, and plastic huts. The interchanging enrichment objects consisted of: whiffle balls, Nyla bones, colored plastic rectangular tubes, colorful plastic key chains, plastic cones, cardboard huts of various shapes, nestlets, colored plastic bowls and cups, and plastic exercise balls. Objects in the enrichment cages were changed every 7 days when cages were cleaned. The enriched groups both received 28 days of enrichment. The enriched removal group was then removed from the enrichment cycle and single-housed 24 h/day with no crinkle paper in the home cage. The continuously enriched group continued enrichment for 14 more days, until sacrifice.

The issue of active cycle enrichment was addressed in our prior study, conducted in males (Smith et al., 2017). In this study we verified that active cycle enrichment replicated the stress profiles of EE and ER groups exposed to continuous enrichment. These data suggest that the physiological and behavioral effects of EE and ER can be replicated in this paradigm. In addition, the inactive cycle is the period of time where the animals are normally sleeping, and thus not likely to experience a negative impact of the lack of enrichment.

### Food Intake and Body Weight

Immediately before housing manipulations began, body weight was recorded and used as a standard to calculate percent change from initial body weight for the duration of the study. Throughout experimentation, body weight was recorded every 7 days. Daily food and water intake, during the inactive period only, was measured for 1 week prior to and for 2 weeks following the enrichment removal.

### Restraint and Blood Sampling

The acute restraint stress test began 1 h into the light cycle near the circadian trough of corticosterone secretion, 7 days post-removal. Rats were placed inside well-ventilated, clear Plexiglas^®^ tubes for 30 min (6.35 cm inner diameter and 20.5 cm length). While in the restrainers, animals were video recorded for later analysis of struggling behavior as an index of helplessness (Atkinson and Waddell, 1997; Sharp et al., 2003). Parameters measured include active struggling; grooming; freezing (noticeable tensing); and still behavior (lack of movement, no noticeable tensing). For assessment of the HPA axis response to acute stress, blood collection was as follows: An animal was placed into the restraint tube and blood was collected by tail clip within 3 min from the initial disruption of the home cage for time 0. The initial tail clip is done by removing 1–2 mm of the distal portion of the tail with a razor blade. Blood samples (approximately 250 (μL) were collected from tail clips by milking blood from into a microcentrifuge tube containing 10 microliters of 100 mM EDTA, and were immediately placed on ice. This method of tail clipping provides for blood collection to occur at all time points without additional tail clipping to be required. At 15, 30, 60, and 120 min from the start of the restraint, blood was also collected. After blood was collected for the 30-min time point, the animal was removed from the restraint and placed back into the home cage. At time points 60 and 120 min, the animal was removed from the home cage and blood samples obtained by gently removing the clot while freely moving. All samples were collected within 3 min. Samples were centrifuged at 3000 ×*g* for 15 min at 4°C, and plasma was stored at –20°C until radioimmunoassay. Females were swabbed to determine estrous cycle at 18:00, approximately 8 h after the start of the restraint.

### Sucrose Preference Test (SPT)

Animals were habituated to two water bottles per home cage during the light cycle for 3 days before administration of sucrose. Habituation started on day 7 and testing was begun on day 10 after enrichment removal in the ER group. Once habituated, rats were given access to a water bottle with (1%) sucrose (Sigma-Aldrich) as well as a standard water bottle for 3 days in their home cages. Positions of the bottles were alternated each day to avoid place preference within the rats. Bottles were weighed prior to administration and after the end of the light cycle duration (11 h). Bottles containing sucrose solution were supplied 1 h after lights on and removed immediately prior to the start of the dark cycle. Data collection included grams of sucrose solution intake and grams of water intake. The percentage of sucrose solution intake per total fluid consumption was used to determine sucrose preference per day for each animal. Females were swabbed to determine estrous cycle at 18:00 following testing on each of the 3 days.

### Forced Swim Test

Rats were exposed to the FST 14 days post-removal. Starting 1h into the lights on period, each rat was placed individually into a Plexiglas cylindrical tank (45 cm H × 20 cm diameter) containing 31 ± 3 cm of water (25 ± 2°C). The rats were monitored for ten minutes before removal from the tank. Behavior during the test was video monitored, with swimming, diving, immobility, and climbing behaviors assessed. The behaviors scored are defined as follows: (i) swimming — moving limbs in an active manner and making circular movements around the tank, (ii) diving-head fully immersed in the water, (iii) immobility — only necessary movements to keep head above water; and (iiii) climbing — rapid movement of limbs up the side of the tank. Animals were killed 85 min after FST initiation by overdose with Pentobarbital. Females were swabbed to determine estrous cycle at the time of kill, and organs removed and weighed.

### Radioimmunoassay

Plasma corticosterone concentrations were measured with ^125^I RIA kit (MP Biomedicals Inc, Orangeburg, NY, United States). Samples were run in duplicate when possible.

### Estrus Cycle

Phase of estrus was assessed by vaginal cytology at time points noted above, using standard histological methods (Becker et al., 2005). Sampling time was late in the subjective day, optimized to detect proestrus (P) phase (1800 h) (Smith et al., 1975)) but less optimal for the estrus (E) phase (being late in E phase and nearing transition to diestrus. Due to these limitations, E and P groups were pooled (as per Egan et al. (2018)) to correspond to a time period where gonadal steroids would be particularly elevated during the time of testing (8 h prior to vaginal swabbing). Similarly, diestrus 1 (D1) and diestrus 2 (D2) groups were pooled to correspond to a time period of reduced gonadal steroid secretion (Butcher et al., 1974; Smith et al., 1975).

### Statistics

Cohort effects in these data were assessed by two-way ANOVA with housing manipulation and cohort as factors. If no cohort effect or interaction existed, data were pooled and analyzed by two-way ANOVA with cycle and housing manipulation as factors. This was the case for the behavioral data, organ weights, food intake and body weight. Due to a significant cohort effect, corticosterone responses to stress were assessed by three-way repeated measures ANOVA, using cohort, housing manipulation and cycle as between-subjects factors and time as the repeating factor (see Supplementary Figure S1). SigmaPlot (Systat Software, San Jose, CA, United States) was used for two-way ANOVAs and Statistica (Statsoft/Dell) was used for three-way ANOVAs. *Post hoc* testing, utilizing Fisher’s Least Significant Difference (LSD), was performed following two-way and three-way ANOVAs to assess the effect of housing manipulation within given time and estrous cycle domains. For all data *p* ≤ 0.05 denotes statistical significance. Due to specific hypotheses having been formed *a priori* on the effects of housing manipulation in females, planned comparisons between groups were performed (Maxwell and Delaney, 1990). The distribution of estrus cycle phase across groups was assessed by Chi-square tests. Outliers in the data sets were determined *a priori* by values that exist outside the mean ± 1.96 times the standard deviation and above the upper quartile + 1.5 times the interquartile range or below the lower quartile – 1.5 times the interquartile range. Data are graphed as mean ± the standard error of the mean (SEM).

## Results

### HPA-Axis Response

**FIGURE 2:**
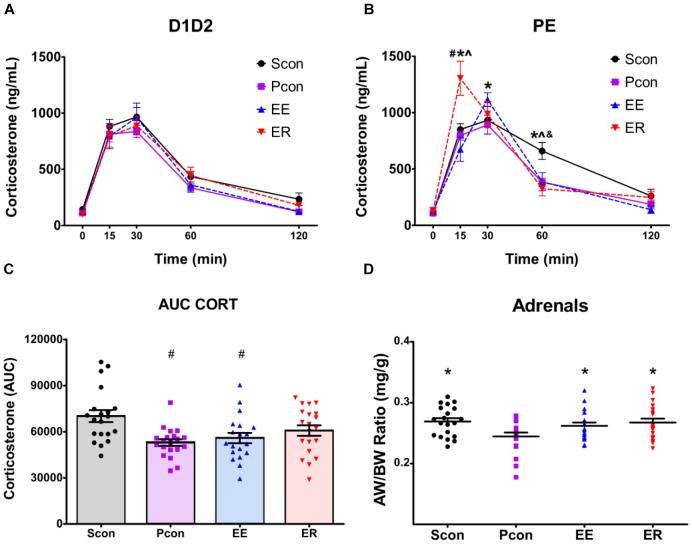
Single-housing (SCon), enrichment (EE), and enrichment removal (ER) in females causes a dysregulation of the HPA axis response to acute restraint stress in a cycle dependent manner and increases adrenal size. Although hormonal time course data for all groups were analyzed by three-way repeated-measures ANOVA (see text), the time profiles of D1D2 and PE animals are shown separately for visual clarity of treatment effects. **(A)** No effects of housing manipulations on stress-induced corticosterone levels were observed for females in the D1D2 stage of the estrous cycle. **(B)** For females in the PE stage of estrous cycle, ER animals have increased corticosterone at 15 min after the start of restraint, EE animals have increased corticosterone at 30 min after the start of restraint, and Scon animals have increased corticosterone at 60 min after the start of restraint ^#^*p* < 0.05 vs. Scon, ^∗^*p* < 0.05 vs. Pcon, ^∧^*p* < 0.05 vs. EE, ^&^*p* < 0.05 vs. ER. **(C)** Total corticosterone response to restraint is increased in the Scon group ^∗^*p* < 0.05 vs. Pcon, ^∧^*p* < 0.05 vs. EE. **(D)** Single-housing, enrichment and enrichment removal in females causes adrenal hypertrophy ^∗^*p* < 0.05 vs. Pcon.

At 7 days post removal of enrichment, the rats were subjected to a 30 min restraint stress in order to measure HPA axis responsiveness in the form of plasma corticosterone levels (CORT) (Figure 2). No differences were observed for amount of time that the animals in any group spent struggling. Analysis of the CORT response to acute restraint showed an increase in corticosterone secretion in all groups following the restraint [main effect of time; *F*_(4,284)_ = 270.32; *p* < 0.001], with levels returning to baseline at 120 min after stressor initiation.

There was a main effect of housing manipulation [*F*_(3,71)_ = 3.0508; *p* < 0.05], an interaction effect of housing manipulation × time [*F*_(12,284)_ = 2.8065; *p* < 0.002] and an interaction effect of housing manipulation × time × estrous cycle [*F*_(12,284)_ = 2.8558; *p* < 0.002]. For the females in the P and E phases, *post hoc* tests revealed that at time 15, the enriched-removed (ER) females had higher (*p* < 0.05) CORT levels compared to the continuously enriched (EE) and both the single (Scon) and pair (Pcon) housed controls. At time 30, the EE females showed higher (*p* < 0.05) CORT levels compared to the Pcon. At time 60, the Scon rats maintained higher (*p* < 0.05) CORT levels than all other animals (Figure 2B). For the females in the P and E phases, there was no effect of housing manipulation on CORT levels at times 0 and 120.

For females within the D1D2 phase of estrous cycle, there were no differences in CORT at any of the time points among the different housing manipulation groups (Figure 2A).

There was a main effect of cohort [*F*_(1,71)_ = 23.228; *p* < 0.001], with cohort 1 having a higher least square mean value than cohort 2, probably related to inter-assay variability in the corticosterone assay. As such, an individual analysis was also conducted for each cohort. The results of the individual analysis and corresponding graphs can be found in the Supplementary Figure S1.

The total corticosterone response to the acute restraint stress, in the form of time-integrated area under the curve (AUC) showed a main effect of housing manipulation [*F*_(3,71)_ = 5.100; *p* = 0.003], no main effect of cycle, and no interaction effect. *Post hoc* tests revealed a significant decrease (*p* < 0.05) in the total CORT response of the continuously enriched and pair housed control females when compared to the single-housed animals (Figure 2C). There was a main effect of cohort [*F*_(1,71)_ = 6.300; *p* = 0.014],with cohort 1 having a higher integrated corticosterone values than cohort 2. As such, an individual analysis was conducted for each cohort. The results of the individual analysis and corresponding graphs can be found in the Supplementary Data.

### Sucrose Preference Test

**FIGURE 3:**
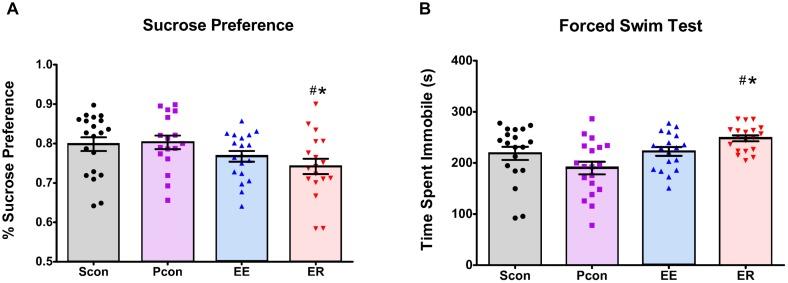
Enrichment removal increases anhedonia in the sucrose preference test and passive coping behavior in the forced swim test. **(A)** ER animals have a decreased preference for sucrose on the first day of the sucrose preference test relative to PCon and Scon groups ^#^*p* < 0.05 vs. Scon, ^∗^*p* < 0.05 vs. Pcon. **(B)** ER females spent significantly more time immobile in the FST relative to pair housed controls (Pcon) and single housed animals (Scon) ^#^*p* < 0.05 vs. Scon, ^∗^*p* < 0.05 vs. Pcon.

Ten days after the enrichment removal, we assessed sucrose preference during the inactive cycle of the rats. Enrichment-removed (ER) animals had a decrease in sucrose preference [*F*_(3,70)_ = 2.766; *p* = 0.048] (Figure 3A) relative to both the single (Scon) and pair (Pcon) housed controls (*p* < 0.05). The continuously enriched (EE) animals showed a decrease in total sucrose intake [*F*_(3,73)_ = 6.069; *p* < 0.001] in comparison to the single (Scon) and pair (Pcon) housed controls (*p* < 0.05). However, the EE females also had a significant decrease in water intake [*F*_(3,73)_ = 8.659; *p* < 0.001] compared to the ER and Pcon females (*p* < 0.05). There was no cohort effect on sucrose preference [*F*_(1,72)_ = 2.303; *p* = 0.133], so the two cohorts were pooled for analysis. There was also no main effect of cycle [*F*_(1,71)_ = 2.319; *p* = 0.132] on the sucrose preference.

### Forced Swim Test

Animals were tested for passive coping behavior in the FST 14 days after enrichment removal. Enrichment-removed (ER) females showed increased time spent immobile [*F*_(3,66)_ = 3.310; *p* = 0.025] (Figure 3B) in comparison to the single (Scon) and pair (Pcon) housed controls (*p* < 0.05). Pair housed control females spent more time swimming [*F*_(3,70)_ = 7.114; *p* = 0.003] than all other females (*p* < 0.05). There were no differences in any other behaviors in the FST. There were also no differences observed in any behaviors during the FST across the estrous cycle [*F*_(1,66)_ = 1.461; *p* = 0.231]. Two-way ANOVA comparison of time spent immobile in the females showed a main effect of housing manipulation [*F*_(3,68)_ = 6.204; *p* < 0.001] but no main effect of cohort [*F*_(1,68)_ = 3.610; *p* = 0.062] and no interaction effect. Based on this, we decided to pool both cohorts for analyses.

### Organ Weights

Somatic effects of enrichment removal were assessed in the form of specific organ weights. Following perfusion, the hearts, thymi, and adrenals of all animals were cleaned, weighed and normalized to animal body weight. There was a main effect of housing manipulation [*F*_(3,73)_ = 3.518; *p* = 0.019] on the adrenals. Single-housed, continuously enriched and enrichment-removed females had larger adrenals per bodyweight compared to the pair-housed controls (*p* < 0.05) (Figure 2D). No effects of housing manipulation were observed on the heart and thymus weights. There was a main effect of cohort [*F*_(1,72)_ = 17.810; *p* < 0.001],with cohort 1 having a higher least square mean than cohort 2. As such, an individual analysis was conducted for each cohort. The results of the individual analysis and corresponding graphs can be found in the Supplementary Table S1.

### Food Intake and Body Weight

**FIGURE 4:**
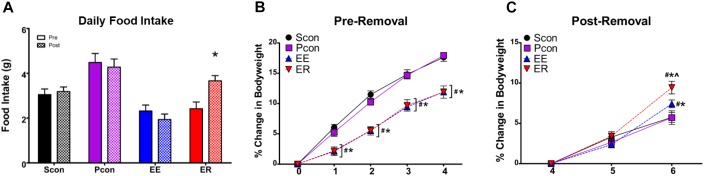
Enrichment removal leads to hyperphagia and an increase in body weight gain. **(A)** Enrichment removal increases inactive cycle food intake ^∗^*p* < 0.05 vs. pre for the ER group only. **(B)** Enrichment leads to an attenuation of body weight gain for both EE and ER groups relative to pair housed controls (Pcon) and single housed animals (Scon) for the first 4 weeks of enrichment ^#^*p* < 0.05 vs. Scon, ^∗^*p* < 0.05 vs. Pcon. **(C)** Rate of change in body weight is increased in the EE group and exaggerated in the ER group at 2 weeks post removal ^#^*p* < 0.05 vs. Scon, ^∗^*p* < 0.05 vs. Pcon, ^∧^*p* < 0.05 vs. EE.

Daily food intake, during the inactive period only, was measured for 1 week prior to and for 2 weeks following the enrichment removal. Comparison of food intake prior to and following enrichment removal within each group showed a main effect of housing manipulation [*F*_(3,76)_ = 12.747; *p* < 0.001], a main effect of time [*F*_(1,76)_ = 4.773; *p* = 0.032], and an interaction effect of housing manipulation × time [*F*_(3,76)_ = 10.390; *p* < 0.001]. *Post hoc* tests revealed that only within the enrichment-removed (ER) group was there a significant increase in food intake following the enrichment removal (*p* < 0.05) (Figure 4A). Note that intake data measures food consumption during the inactive period only.

Body weight measurements were taken weekly and reported as the percentage of change from the original body weight of the animal prior to starting the housing manipulations. Analysis of body weight change during the first 4 weeks of the study showed a main effect of housing manipulation [*F*_(3,299)_ = 22.877; *p* < 0.001], a main effect of time [*F*_(4,299)_ = 996.525; *p* < 0.001], and an interaction effect of housing manipulation × time [*F*_(12,299)_ = 13.050; *p* < 0.001]. *Post hoc* tests revealed that starting 1 week into enrichment and until the time of enrichment removal, EE and ER animals had a significant attenuation of body weight gain compared to the Scon and Pcon animals (*p* < 0.05) (Figure 4B). Analysis of body weight at the time of enrichment removal, as well as once weekly for the next 2 weeks, showed a main effect of housing manipulation [*F*_(3,152)_ = 14.612; *p* < 0.001], a main effect of time [*F*_(2,152)_ = 295.660; *p* < 0.001], and an interaction effect of housing manipulation × time [*F*_(6,152)_ = 6.026; *p* < 0.001]. *Post hoc* tests revealed at the time of enrichment removal, as well as 1-week post, the continuously enriched (EE) and enrichment-removed (ER) females had an attenuation in body weight gain compared to the single (Scon) and pair (Pcon) housed controls (*p* < 0.05). At 2-weeks post removal, only the EE group was showing attenuated body weight gain compared to the single (Scon) and pair (Pcon) housed controls (*p* < 0.05) (Figure 4C). To understand the rate of change in body weight after enrichment removal in the same fashion as in the males (Smith et al., 2017), we calculated the percent change in body weight beginning at the point of enrichment removal in all groups. There was a main effect of housing manipulation on body weight change [*F*_(3,152)_ = 3.643; *p* = 0.016], a main effect of time [*F*_(2,152)_ = 295.660; *p* < 0.001], and an interaction effect of housing manipulation × time [*F*_(6,152)_ = 6.026; *p* < 0.001]. *Post hoc* tests revealed no differences in the rate of body weight gain 1 week after removal. At 2 weeks post removal, the ER group had a significant increase in weight gain compared to all other groups (*p* < 0.05) and the EE group had a significant increase in weight gain compared to the Scon and Pcon animals (*p* < 0.05) (Figure 4C).

### Estrous Cycle

We monitored phase of estrus cycle at the conclusion of behavioral testing sessions to allow determination of the possible impact of housing conditions on cyclicity. As would be expected, the number of animals in diestrus was significantly greater than that of proestrus and estrus (Table 1), and this distribution did not differ with treatment condition (Table 2) (Chi-square).

**Table 1:** Number of animals in either D1/D2 or P/E on days of testing.

Cycle:	FST (10AM)	Restraint bleed (6PM)	Sucrose preference (Day One) (6PM)	Sucrose preference (Day Two) (6PM)	Sucrose preference (Day Three) (6PM)
D1/D2	56	44	37	49	56
P/E	22	25	42	31	24
Undetermined	2	1	1	0	0

**Table 2:** Number of animals in either D1/D2 or P/E across experimental groups.

Cycle:	Treatment:	FST (10AM)	Restraint bleed (6PM)	Sucrose preference (Day One) (6PM)	Sucrose preference (Day Two) (6PM)	Sucrose preference (Day Three) (6PM)
D1/D2	Single-Housed	13	10	9	16	12
	Pair-Housed	15	11	9	11	15
	EE	14	11	10	8	13
	ER	14	12	9	14	16
P/E	Single-Housed	7	10	11	4	8
	Pair-Housed	4	9	10	9	5
	EE	6	9	10	12	7
	ER	6	8	11	6	4

## Discussion

Our studies indicate that loss driven by enrichment removal increases passive coping behavior and hyperphagia during the inactive cycle. These results recapitulate findings in males and suggest that loss engenders similar adaptations in both sexes. However, unlike males, enrichment removal decreases sucrose preference in females, suggesting that loss has sex-specific effects on reward circuits in the brain.

Enrichment removal increased immobility and decreased active behaviors in the FST relative to pair housed controls and single-housed animals, suggesting that the experience of loss selectively increased passive coping behavior (Commons et al., 2017; Smith et al., 2017). Immobility in the FST is interpreted by some as helplessness behavior, and can thereby linked to depressive symptomology [however, this is not the consensus interpretation (see de Kloet and Molendijk, 2016)]. This contention is made in the context of testing antidepressant drugs but is more difficult to apply as a “phenotyping tool” in the absence of antidepressant therapy here. However, it is important to note that in males, increased FST immobility following ER is reversed by antidepressant (imipramine) treatment (Smith et al., 2017), and thus the observed behavioral change may be consistent with alterations in mood.

Removal from enrichment differentially increased rate of body weight gain and food intake relative to other groups, an observation also seen in males. These data suggest that ER has a lasting impact on metabolic regulation, perhaps via modulation of central processes regulating food intake or energy expenditure. In ER males, increased body weight was accompanied by increases in non-homeostatic feeding (Smith et al., 2017)). Notably, stress is known to contribute to overeating, particularly of highly palatable calorically dense foods (Dallman et al., 2005; Ulrich-Lai et al., 2015; Packard et al., 2017), and these data suggest that ER may reflect stress facilitation of food intake. Continuously enriched females also show an increased rate of body weight gain compared to the pair housed controls and the single-housed animals. Although the data suggest no increase in daily food intake for these animals, it is important to note that due to the logistics of the study food intake was only measured during the inactive period for all animals and as such we may have missed any changes in food intake during the active period. Importantly, stress-induced eating has not been reproducibly observed in rodents exposed to most imposed stress regimens, such as social defeat, chronic variable stress, chronic restraint, etc. [e.g., see Harris, 2015; Herman et al., 1995; Tamashiro et al., 2004]. It is notable that chronic social stress regimens produce stress-related hyperphagia and weight gain in subordinate mice (Bartolomucci et al., 2004). Thus, the experience of loss may tap into physiological or psychological processes linking social stressors to ingestive behavior.

Despite the fact that enrichment-removed females show increased daily food intake, assessment of sucrose intake indicate reduced sucrose preference in ER females vs. both pair-housed controls and single-housed groups. These data are consistent with augmented anhedonic behavior following ER. Although full interpretation of these findings are limited by the inability to reliable assess food intake during enrichment periods, the data suggest that consequences of ER on hedonic processes may differ substantially in males and females, perhaps dictated by differential effects of ER on central reward pathways.

Female HPA axis responses to restraint revealed several effects of housing manipulations on plasma corticosterone (CORT) levels. Interestingly, all of the housing effects were observed only in the PE stages (and not D1D2 stages) of the estrous cycle at the time of the acute stressor. Continuously enriched females show a heightened peak CORT response compared to the pair-housed controls, which is reminiscent of the effect reported in males (Smith et al., 2017). However, enrichment removal in males leads to a blunted peak response (Smith et al., 2017), whereas in females, it drives an early and exaggerated peak response in the PE stage. Additionally, single housing delays CORT recovery, suggesting that social isolation is sufficient to alter HPA axis feedback in females. Given that estradiol levels are high in both P and E phases (Becker et al., 2005), these data suggest that housing conditions interact with HPA axis reactivity in an estrogen-dependent manner. The prolonged response observed in single-housed animals during P and E phase corresponds to the period of sexual receptivity, which perhaps intensifies the experience of individual housing (Becker et al., 2005).

Adrenal weights were increased in all experimental groups vs. pair-housed controls. Adrenal weight is determined in large part by history of ACTH release, and increases are generally thought to reflect a hyperactive HPA axis (Herman et al., 2016). Consequently, it appears that EE, ER, and single housing all produce some degree of prolonged HPA axis drive relative to the primary control group (pair housed). Enhanced HPA axis reactivity for all of these housing manipulations is supported by the observed increases in CORT mentioned above. These data add to a growing literature noting that females may be particularly susceptible to “stressful” effects of single housing or social isolation (Vieira et al., 2018). Increased adrenal weight in the continuously enriched group may also be related to increased need for glucocorticoid secretion during periods of high activity in the enrichment cages. Consistent with this possibility, it is known that voluntary exercise in male rats induces adrenal hypertrophy and increased corticosterone response to a forced swim stress (Droste et al., 2007). Thus, additional studies would need to be done to isolate which, if not both, enrichment or the subsequent social isolation is causing the adrenal hypertrophy in enrichment-removed females.

Estrus cycles were monitored at the conclusion of behavioral and HPA axis testing, to check for any stage effects on experimental endpoints. There was no effect of estrus stage on behavior in the FST or sucrose preference tests, suggesting that the behaviors tested were not sensitive to hormonal variations across the cycle. Moreover, none of the housing manipulations appear to affect estrus cycling, as there were no differences in the proportion of animals in D1D2 or PE at any point in the experiment (However, it is important to acknowledge that the design was not optimized to clearly differentiate individual stages of estrus, and thus our interpretation is limited to periods likely to have elevated (PE) or low (D1D2) estradiol and progesterone secretion during testing).

Continuous active-cycle enrichment does not recapitulate the effects of ER on passive coping and sucrose preference, indicating that the core findings are associated with the removal of enrichment, not the act of enrichment itself. Active cycle EE does not appear to deviate significantly from either single-housed animals or pair-housed controls in terms of passive coping, sucrose preference or daily food intake. The EE group does show an increased rate of body weight gain compared to the Scon and Pcon females but this effect is attenuated compared to the ER group. However, it is useful to note that the EE group drank less sucrose and water during the inactive cycle (with the same preference ratio). Rodents take in the majority of their daily calories and water during the active cycle, and it is possible that the lower raw intake level may be related to decreased activity or increased sleep during the inactive period (perhaps due to the high level of activity in EE period) (Abou-Ismail et al., 2010).

Overall, the data from the study support the usefulness of enrichment removal to induce behavioral symptoms associated with loss in humans, including passive coping, anhedonia and altered eating patterns. Importantly, ER causes symptoms consistent with negative mood in both males and females, indicating its utility for probing neurobiological underpinnings of loss-related dysphoria and dysfunction. Future studies are needed to explore the neural mechanisms and circuits that underlie loss phenotypes.

## Author Contributions

RM, OH, BS, and JH designed the experiments and wrote and edited the manuscript. RM and OH performed the experiments and analyzed the data.

## Conflict of Interest Statement

The authors declare that the research was conducted in the absence of any commercial or financial relationships that could be construed as a potential conflict of interest.
