# Supplementary material for: Loss of Environmental Enrichment Elicits Behavioral and Physiological Dysregulation in Female Rats
Source: Front Behav Neurosci. 2019 Jan 21;12:287. doi: 10.3389/fnbeh.2018.00287 (PMC6357926; doi:10.3389/fnbeh.2018.00287)
Supplement: Supplementary file 1 [file Data_Sheet_1.PDF]

Supplementary Data

Study Design: Cohorts

Cohort refers to the acclimation time (5 days vs. 7 days) and group refers to the housing manipulation. Within each housing manipulation (n=20) 10 rats were from the first cohort (5 days) and 10 rats were from the second cohort (7 days). Each cohort experienced the same experimental timeline but the start of the timeline was offset by 2 days between cohorts to allow each cohort to be tested separately. The experimental timeline for the second cohort began two days after the experimental timeline for the first cohort had begun. Decreasing the number of animals tested per day allowed for the testing time for the restraint and FST to be done closer to the circadian corticosterone trough and thus minimizing the fluctuations in diurnal hormone levels at the time of testing. [Helen C. Atkinson, Brendan J. Waddell; Circadian Variation in Basal Plasma Corticosterone and Adrenocorticotropin in the Rat: Sexual Dimorphism and Changes across the Estrous Cycle, Endocrinology, Volume 138, Issue 9, 1 September 1997, Pages 3842–3848, https://doi.org/10.1210/endo.138.9.5395].

Supplementary Table 1: Description of Cohorts

Scon	Pcon	EE	ER
Cohort 1 (5 days)<br>n=10	Cohort 1 (5 days)<br>n=10	Cohort 1 (5 days)<br>n=10	Cohort 1 (5 days)<br>n=10
Cohort 2 (7 days)<br>n=10	Cohort 2 (7 days)<br>n=10	Cohort 2 (7 days)<br>n=10	Cohort 2 (7 days)<br>n=10

HPA-axis Response

At 7 days post removal of enrichment, the rats were subjected to a 30 min restraint stress in order to measure HPA axis responsiveness in the form of plasma corticosterone levels (CORT). There was a main effect of cohort [$F(1,71)=23.228$; $p=0.00001$], with cohort 1 having a

higher least square mean value than cohort 2. As such, an individual analysis was conducted for each cohort. **Cohort 1:** Analysis of the CORT response to acute restraint showed an increase in the mean corticosterone levels of all animals following the restraint [main effect of time; $F_{(4,128)}=226.71$; $p<0.001$] with levels returning close to baseline at 120 minutes after stressor initiation.

There was a main effect of housing manipulation [$F_{(3,32)}=4.2057$; $p=0.01288$] and an interaction effect of housing manipulation x time [$F_{(12,128)}=4.0327$; $p=0.00003$]. There was no main effect of cycle, and no interaction effects of housing manipulation x cycle, time x cycle or housing manipulation x time x cycle. Post hoc tests revealed that at time 15, the enriched-removed (ER) females had higher ($p<0.05$) CORT levels compared to the continuously enriched (EE) and both the single (Scon) and pair (Pcon) housed controls. At time 30, the EE females showed higher ($p<0.05$) CORT levels compared to the Pcon. Also at time 30, the Scon females showed higher ($p<0.05$) CORT levels compared to the ER and Pcon. At time 60, the Scon rats maintained higher ($p<0.05$) CORT levels than all other animals. There was no effect of housing manipulation on CORT levels at times 0 and 120 (S1A).

The total corticosterone response to the acute restraint stress, in the form of time-integrated area under the curve (AUC) showed a main effect of housing manipulation [$F_{(3,32)}=5.177$; $p=0.005$], no main effect of cycle and no interaction effect. Post hoc tests revealed a significant increase ($p<0.05$) in the total CORT response of the single-housed animals compared to all other groups (S1B).

Cohort 2: Analysis of the CORT response to acute restraint showed an increase in the mean corticosterone levels of all animals following the restraint [main effect of time; $F_{(4,124)}=120.45$; $p<0.001$] with levels returning close to baseline at 120 minutes after stressor initiation.

There was no main effect of housing manipulation but there was a significant interaction effect of housing manipulation x time [$F_{(12,124)}=3.3303$; $p=0.00033$]. There was a main effect of

cycle [$F_{(1,31)}$=6.8400; p=0.01365], no interaction effects of housing manipulation x cycle or time x cycle. There was an interaction effect of housing manipulation x time x cycle [$F_{(12,124)}$=2.3051; p=0.01091]. Although hormonal time course data for all groups was analyzed by three-way repeated-measures ANOVA, the time profiles of D1D2 and PE animals are discussed separately for clarity of treatment effects. Post hoc tests revealed that at time 15 for females within the D1D2 cycle stage, the enriched (EE) and the enriched-removed (ER) females had lower ($p<0.05$) CORT levels compared to the single (Scon) and pair (Pcon) housed controls. No effects of housing manipulation were observed at any other time points for the D1D2 females (S2A).

For females in the PE stage of estrous cycle, ER animals have increased ($p<0.05$) corticosterone compared to the EE and Scon animals at 15 mins after the start of the restraint. Additionally, the EE females at time 15 have lower ($p<0.05$) CORT than all other groups. At time 30, the EE and ER females showed higher ($p<0.05$) CORT levels compared to the Scon. At time 60, the Scon rats maintained higher ($p<0.05$) CORT levels than the Pcon animals. There was no effect of housing manipulation on CORT levels at times 0 and 120 (S2B).

The total corticosterone response to the acute restraint stress, in the form of time-integrated area under the curve (AUC) showed no main effect of housing manipulation, no main effect of cycle and no interaction effect (S2C).

Organ Weights

Somatic effects of enrichment removal were assessed in the form of specific organ weights. Following perfusion, the adrenals of all animals were cleaned, weighed and normalized to animal body weight. There was a main effect of cohort [$F_{(1,72)}$=17.810; p<0.001],with cohort 1 having a higher least square mean than cohort 2. As such, an individual analysis was conducted for each cohort. **Cohort 1:** There was a main effect of housing manipulation [$F_{(3,36)}$=3.174;

p=0.036] on the adrenals. Single-housed females had larger adrenals per bodyweight compared to the continuously enriched and pair-housed controls ($p < 0.05$). Enrichment removed females had larger adrenals per bodyweight compared to the pair-housed controls ($p < 0.05$) (S3A).

Cohort 2: There was a main effect of housing manipulation [$F_{(3,36)}=3.117$; p=0.038] on the adrenals. Single-housed, continuously enriched and enrichment removed females had larger adrenals per bodyweight compared to the pair-housed controls ($p < 0.05$) (S3B).

Discussion

HPA axis profiles and adrenal weights (generally taken as a long-term index of HPA axis activation (ACTH release)) appear qualitatively similar across groups in the two cohorts, but inclusion of cohort as a variable did reveal significant differences as noted above. In the case of corticosterone determinations, these differences may be a result of earlier testing of cohort 1 (5 days post arrival in the vivarium vs. 7 days), and appeared to be carried largely by the single-housed group. Cohort 1 also exhibited larger overall adrenal size, perhaps indicative of a lasting effect of vivarium acclimatization that permeated all groups. Whereas the data generally support the impact of housing mamipulations in both cohorts, the data raise the alraming possibility that acclimation period has lasting effects on stress-related endpoints in female rats, and that these may be particularly robust when females are singly housed.

In contrast with HPA endpoints, no cohort effects were evident on body weight/food intake or behavioral tests. These data suggest that physiological responses may be particularly prone to the impact of adaptation to shipping/new housing conditions.

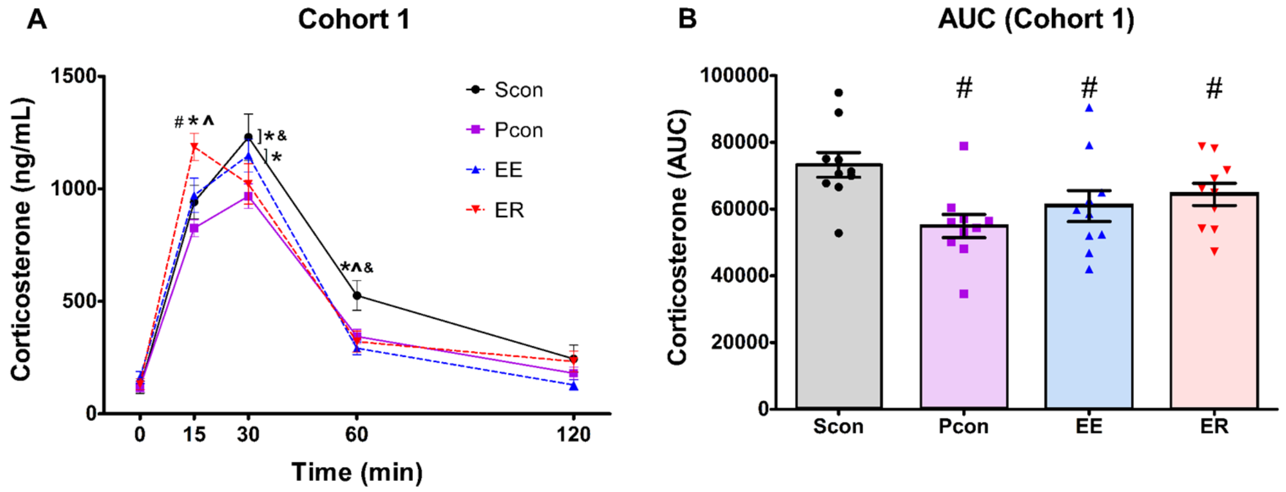

Supplementary Fig. 1: Single-housing, enrichment and enrichment removal in females of cohort 1 display a dysregulation of the HPA axis response to acute restraint stress independent of estrous cycle stage. **(A)** ER animals have increased corticosterone at 15 mins after the start of restraint, EE and Scon animals have increased corticosterone at 30 mins after the start of restraint, and Scon animals maintain increased corticosterone at 60 mins after the start of restraint $^{\#}p<0.05$ vs Scon, $^{*}p<0.05$ vs Pcon, $^{\wedge}p<0.05$ vs EE, $^{\&}p<0.05$ vs ER. **(B)** Total corticosterone response to acute restraint is increased in the Scon group $^{\#}p<0.05$ vs Scon.

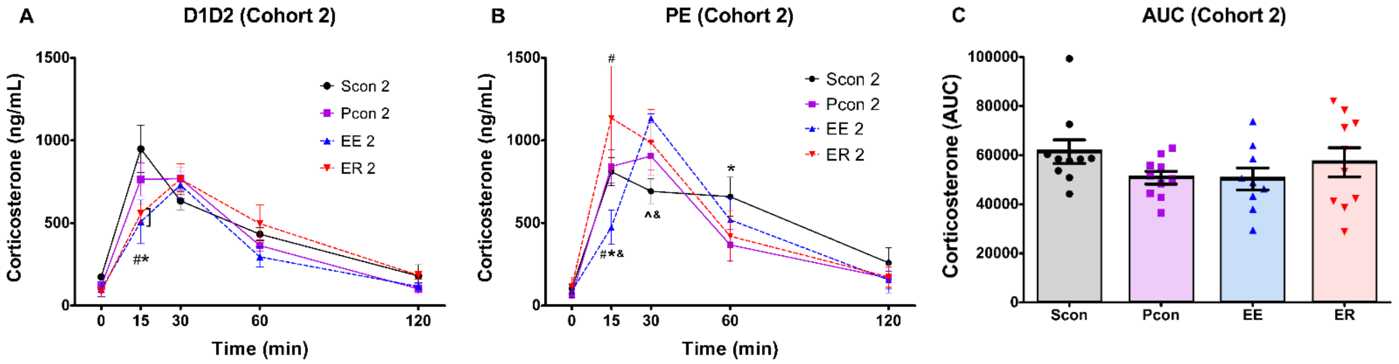

Supplementary Fig. 2: Single-housing, enrichment and enrichment removal in females of cohort 2 leads to a dysregulation of the HPA axis response to acute restraint stress in a cycle dependent manner. Although hormonal time course data for all groups was analyzed by three-way repeated-measures ANOVA, the time profiles of D1D2 and PE animals are shown separately for clarity of treatment effects. **(A)** 15 minutes from the start of restraint, enrichment and enrichment removal attenuate stress-induced corticosterone levels in females in the D1D2 stage of the estrous cycle. **(B)** For females in the PE stage of estrous cycle, ER animals have increased while EE animals have decreased corticosterone at 15 mins after the start of restraint. EE and ER animals have increased corticosterone at 30 mins after the start of restraint, and Scon animals maintain increased corticosterone at 60 mins after the start of restraint $^{\#}p<0.05$

vs Scon, *p<0.05 vs Pcon, ^p<0.05 vs EE, &p <0.05 vs ER. **(C)** Total corticosterone response to restraint is not affected by housing manipulation in cohort 2.

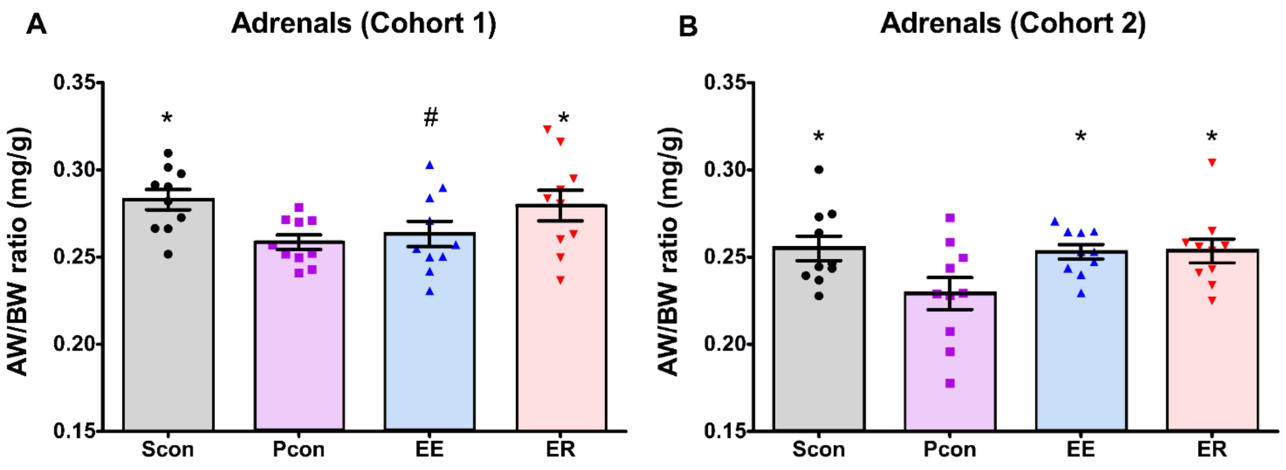

Supplementary Fig. 3: Single-housing, enrichment and enrichment removal in females leads to adrenal hypertrophy. **(A)** In cohort 1, singly-housed females display adrenal hypertrophy compared to continuously enriched and pair housed control females. Enrichment removal females only show adrenal hypertrophy in comparison to the pair housed controls #p<0.05 vs Scon, *p<0.05 vs Pcon . **(B)** For females in Cohort 2, single-housing, enrichment and enrichment removal all display adrenal hypertrophy compared to the pair housed controls *p<0.05 vs Pcon.
